# A Diagnostic Insight of Dental Pulp Testing Methods in Pediatric Dentistry

**DOI:** 10.3390/medicina58050665

**Published:** 2022-05-16

**Authors:** Andreea Igna, Doina Mircioagă, Marius Boariu, Ștefan-Ioan Stratul

**Affiliations:** 1 Department of Pediatric Dentistry, Pediatric Dentistry Research Center, Faculty of Dental Medicine, “Victor Babeș” University of Medicine and Pharmacy, 300041 Timișoara, Romania; 2 Department of Physical Education, University Sport Research Center for Evaluation of Fitness Level—CUSENF, “Victor Babeș” University of Medicine and Pharmacy, 300041 Timișoara, Romania; doina.mircioaga@umft.ro; 3 Department of Endodontics, Faculty of Dental Medicine, TADERP Research Center, “Victor Babeș” University of Medicine and Pharmacy, 300041 Timișoara, Romania; 4 Department of Periodontology, Faculty of Dental Medicine, Anton Sculean Research Center for Periodontal and Peri-Implant Diseases, “Victor Babeș” University of Medicine and Pharmacy, 300041 Timișoara, Romania; s.stratul@gmail.com

**Keywords:** pulp diagnosis, primary teeth, immature permanent teeth, sensibility tests, vitality tests, pediatric dentistry

## Abstract

The accurate diagnosis of pulpal pathology in pediatric dentistry is essential for the success of vital pulp therapy. Pulp testing is often a challenging task due to understanding and cooperation issues of pediatric patients, as well as the particularities of pulpal physiology encountered in primary and immature permanent teeth. Sensibility tests, although still widely used by dental practitioners, are no longer recommended by pediatric specialists mainly due to their subjective nature. Vitality pulp tests have gained popularity in the last decade in light of some encouraging results of clinical studies. However, their use is not a routine practice yet. This paper is a literature review aimed to guide dental practitioners towards selecting the appropriate pulp testing method for their pediatric cases. It provides an overview on a multitude of pulp testing methods and an update in recommendations for primary and immature permanent teeth.

## 1. Introduction

Deep dental caries and dental trauma to the primary and immature permanent dentition are frequent occurrences in a pediatric dental practice, and they present a challenge concerning the evaluation of the pulpal status and consequent choice of treatment strategy. In today’s pediatric dentistry, the conservative or minimally invasive dentistry (MID) concept has revolutionized the approach to caries excavation and diagnosis in both primary and permanent teeth. The treatment paradigm has shifted towards techniques aimed at maintaining pulp vitality, allowing the tooth to heal [1], and for that reason, an accurate diagnosis of the pulp status prior to the treatment is essential.

**Figure 1:**
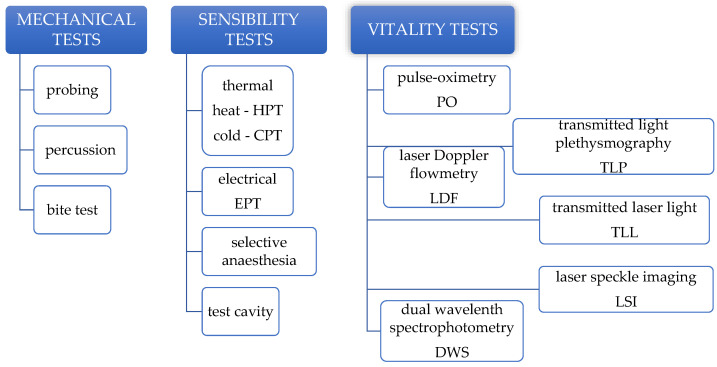
Classification of pulp testing methods.

The accuracy of a pulp diagnosis relies on a combination of data obtained from clinical examination, corroborated with radiographic findings, results of pulp testing, and reported dental history. The diagnostic process is also influenced to some degree by the clinician’s experience in the field [2]. Dental pulp testing is a useful and essential diagnostic aid widely used in endodontics [3]. There are multiple methods of testing the pulp status (Figure 1): either by assessing the neural component of the pulp (triggering a sensory response through mechanical or sensibility tests), or by assessing the vascular component (through vitality tests). The use of sensibility tests in primary and in immature permanent teeth to evaluate pulp status is frequently a challenging task, due to the particularities in the pulp physiology of these teeth and to the fact that the results of the tests are highly subjective, depending on the patient’s cooperation and understanding of the situation, which is particularly difficult in young children [4]. Mechanical tests are equally challenging in children, as any pain stimulation may hinge cooperation for further treatment. Vitality tests, on the other hand, proved to be more reliable, patient-friendly (pain-free), and objective, but they still come with a few drawbacks that have prevented them from being routinely used in pediatric practice.

The ideal technique for the evaluation of the dental pulp status needs to be non-invasive, objective, painless, reliable, reproducible, and standardized [5]. To this list, we can add two more requirements that are essential for pediatric use: easy to employ (a simple technique) and fast. The diagnostic accuracy of a pulp test is measured using a gold or reference standard for comparison. The gold standard is the best available method against which the performances of other diagnostic tests are evaluated, whereas a reference standard does not necessarily identify the target condition with 100% accuracy. In the case of the dental pulp, histological examination has been regarded as the gold standard, whereas direct visualization has been used most often as a reference standard in clinical studies [6,7]. In clinical day-to-day practice, the results of pulp testing of intact, healthy adjacent and/or contralateral teeth to the ones in question are often used as controls, to observe a baseline normal response [8]. Teeth with evidence of root canal filling are also used as controls to confirm nonvital teeth [9]. Specificity and sensitivity are two parameters used to assess the outcome of a pulp test, showing its intrinsic ability to correctly identify vital pulp and necrotic pulp, respectively. Specificity refers to the percentage of teeth correctly identified as vital, and sensitivity refers to the percentage of teeth correctly identified as nonvital [10].

## 2. Aspects of Dental Pulp Physiology in Children

A basic structural difference between primary and permanent teeth lies in the thickness of the hard dental structures covering the pulp chambers, which is lower in primary teeth than in permanent teeth. The pulp is correspondingly closer to the outer surface and more susceptible to infection, due to a more rapid progression of dental caries through the thinner tissues [11].

The pulp tissue in primary and permanent teeth exhibit histological and functional similarities. An odontoblastic layer outlines the periphery of the pulp chamber, extending their cytoplasmic processes into the dentinal tubular structure. A cell-free zone is located just below the odontoblastic layer and contains an extensive plexus of nerves—Raschkow’s plexus and blood capillaries that may support and regulate odontoblast activity and provide sensory innervation to the pulp. Raschkow’s plexus is not completely formed until the late stages of root development, which has implications especially for the diagnosis of pulp vitality in immature permanent teeth [12]. The pulp of mature primary teeth is well innervated and has many nerve endings terminating in, or near the odontoblast layer, with a small number penetrating into the dentine [13]. The pattern of the deciduous innervation shows some similarities to the permanent dentition, but a key difference lies in the high density of dentinal innervation found in the cervical region of primary teeth [14]. Based on the results of an in-vitro study on the innervation of primary teeth [15], it seems that there are significant differences between the nerve fibers of first and second molars. In a clinical context, this finding suggests a lower sensitivity to pain of the first primary molars, compared to the second ones, which may have implications for the interpretation of pulp sensibility testing results.

In the face of aggression, the dentin–pulp complex response in primary teeth is also similar to that of permanent teeth, including a reduction in the number of odontoblasts and an increase in the number of inflammatory cells [11].Throughout their lifetime, from development to exfoliation, primary teeth undergo functional and structural changes. An investigation led by Monteiro et al. of the physiological root resorption in primary teeth has revealed that although some changes do occur in the pulpal status of primary teeth within the resorption stage, these are not as profound as previously thought, therefore speculating that teeth could retain the potential for sensation, healing, and repair until advanced stages of root resorption [16].

## 3. Mechanical Pulp Tests

The probing, percussion, and bite tests are mechanical tests used in clinical practice for various diagnostic purposes [3]: to evaluate sensitivity (probing), or to check for inflammatory pathology (bite test, percussion). In pediatric dentistry, such procedures are generally contraindicated [11] because they are invasive and have a high chance of triggering pain, posing the risk of inducing disruptive behavior in an otherwise cooperative child [17]. Instead, palpation of the muco-buccal fold, mobility assessment, and a modified percussion technique can be performed in children. Fluctuation felt by palpation is indicative of an acute dentoalveolar abscess before exteriorization, whereas bone destruction felt by palpation can be a sign of chronic dentoalveolar abscess [18]. Teeth with varying degrees of pulpal inflammation may present a slight mobility, but in primary teeth, mobility can also be a sign of active physiologic root resorption. Comparing the mobility of a suspicious tooth with its ipsilateral equivalent can be helpful for differential diagnosis [12]. When performing percussion in young children, it is indicated to use the tip of the finger (rather than the end of a metal instrument) to apply a gentle pressure on the tooth, in combination with the tell-show-do behavior management technique. The test should begin with a contralateral unaffected tooth to familiarize the patient with a normal response to the stimuli [19]. Sensitivity to percussion is indicative of an acute peri-radicular periodontitis [12].

## 4. Sensibility Pulp Tests

Sensibility is defined as the ability to respond to a stimulus. The results of sensibility pulp tests are essentially qualitative sensory manifestations extrapolated to estimate the “vitality” and state of pulp health [3]. Depending on the nature of the response (pain characteristics), there are three types of likely responses: a mild pain that goes away once the stimulus is removed, which indicates a healthy pulp, an exaggerated, lingering pain (of various duration), which indicates an inflamed pulp (reversibly/irreversibly), and the absence of pain, which is usually associated with pulp necrosis or previous root canal therapy [20]. A study investigating the correlation of symptoms with the histological appearance of the pulp demonstrated that the classification of pulp conditions as normal, reversible, and irreversible pulpitis (based on treatment prognosis) has high chances of guiding the correct therapy in most cases, especially the ones with normal pulp or reversible pulpitis [21]. In the case of multirooted teeth, however, the possibility that a tooth may have necrotic and vital pulp coexisting within the same root canal system must be considered [22].

### 4.1. Thermal Pulp Testing

Thermal pulp testing implies the application of chemical agents on the surface of the teeth to increase (heat pulp testing—HPT) or to decrease (cold pulp testing—CPT) temperature, thus stimulating pulp sensory responses through thermal conduction. These methods have been the most commonly used for pulp testing by general dental practitioners and endodontists [3]. Therefore, we should be aware that not all pulp testing agents and/or methods are suitable for all clinical situations. Sometimes, a combination of pulp tests is used to provide more reliable results [2,23]. In this regard, we can take the study of Peters et al., who obtained statistically significant false positive responses to the cold-testing of multi-rooted teeth (with necrotic and vital pulp, coexisting within the root canal system) [22].

The cold pulp test is the most commonly used pulp test by dental practitioners, including pediatric dentists [24], which provides information about the pulp status (“vital/non-vital”) or inflammation status (reversible/irreversible). Ethyl chloride, ice, CO_2_ snow and refrigerant sprays (tetrafluoroethane, dichlorodifluoromethane or propane/butane/isobutane gas mixture stored in pressurized cans) are a few examples of CPT agents. Ice is the simplest and cheapest agent, which can be obtained by freezing water in bar-shaped silicone molds. The direct application of an ice bar onto a tooth can be uncomfortable though due to the large contact area, and risky because of the possibility of accidentally stimulating multiple teeth. CO_2_ snow is easy to use and safe, but more costly and less reliable [3]. Refrigerant sprays have the advantages that they require no special storage conditions and allow a more precise application using a cotton pellet as a carrier. They have proven effectiveness in both natural teeth and teeth with full coverage restorations [25]. Due to the ease of storage, relatively low cost, and simple application technique, refrigerant sprays are today the most widely used CPT agents in clinical settings [3]. For a reliable response, teeth need to be dried and well isolated [8]. A control tooth should be tested first, so that the patient knows what a normal sensation feels like. It has been shown that, if used correctly, CPT does not pose any risk of injuring the pulp [26].

The heat pulp test (HPT) can be performed either by application of heated water (hot water bath), gutta-percha or compound material heated to melting temperature onto the tooth being tested (using lubricant in order to facilitate removal of the material), or by placing near the tooth (without touching the tooth surface) a heated ball-ended instrument or a battery-powered controlled heating instrument [3,27]. There are a few safety concerns for the use of a heat test in pediatric patients related to increased anxiety triggered by the proximity of a hot instrument (which may cause unwanted events like injuries to the soft tissues) and the possibility of pulp damage (since pulp horns are relatively superficial in deciduous teeth) [28].

### 4.2. Electric Pulp Testing

Electric pulp testing (EPT) consists of the application of an electric stimulus onto the tooth, in the attempt to stimulate the pulpal nerve fibers related to pain and elicit a response from the patient. Although widely used, EPT is highly technique sensitive. There are a few essential requirements needed to obtain reliable results: adequate stimulus, optimal placement of the tester electrode, proper tooth isolation, ensuring a conducting media, appropriate application method and careful interpretation of the results [20]. The value of EPT in assessing the pulp status of primary teeth is viewed with reserves by pediatric dentists. Although EPT provides data about whether a tooth is “vital/non-vital”, it does not provide reliable evidence of the degree of pulpal inflammation and cannot be used to differentiate reversible pulpitis from an irreversible one [29], unlike the CPT. A complicating factor is the occasional false-positive response to the test in teeth with necrotic pulp when the content of the canals is liquid [30]. An increased sensory response threshold to EPT can be present in teeth with intra-pulpal calcification, in patients undergoing orthodontic treatment, and in patients suffering from primary hyperthyroidism, in which cases the sensory response may be completely blocked or may require a much higher current dose to elicit a response from a healthy pulp [31].

### 4.3. Selective Anaesthesia and Test Cavity

Selective anaesthesia is a diagnostic procedure aimed at localizing the source of an acute pain by progressively excluding dental territories until the causative tooth is identified [32]. Although useful in adults, the technique is inappropriate for use in pediatric dentistry. The procedure requires the complete awareness and cooperation of the patient, whereas in younger children, the success of local anaesthesia administration is highly dependent on distraction through behaviour management techniques. Older children may be able to comprehend the procedure, but the repetitive stimulus raises anxiety concerns.

Test cavity is regarded as the last resort for assessing the pulp status of a tooth, because of its invasive and irreversible nature [33]. It is performed by drilling through the enamel-dentine junction of an unanesthetized tooth and asking the patient to signal any pain felt during the procedure. Because of its invasive nature and potential for false-positive responses due to increased anxiety, the test cavity technique is generally avoided in adult patients, and even more so in pediatric patients. Furthermore, it adds no further information beyond what other pulp sensibility tests can provide [33].

### 4.4. Reliability of Sensibility Pulp Tests in Children

Sensibility pulp testing in children, although deemed reliable by some studies [34,35,36,37], highly depend on the child’s apprehension associated with the test itself [38]. In addition, invalid data might be obtained as a result of the often unreliable responses from children because of fear, management problems, and inability to understand or communicate accurately [17,39]. Most children perceive sensibility pulp testing as unpleasant stimuli, therefore, false-positive and false-negative responses are commonly encountered. Such situations may lead to incorrect treatment strategies [28,40]. Teeth erroneously diagnosed as nonvital may undergo unnecessary endodontic treatments, whereas those erroneously diagnosed as vital may be left untreated, causing further complications to the supporting tissues [27], and even affecting the successor tooth bud [41] if primary teeth are involved.

In immature permanent teeth, there is a universal agreement that they have a great potential to heal after trauma or deep carious lesions but also the greatest chance to be misdiagnosed or mistreated [39]. Sensibility tests are considered unreliable during the root formation period, due to the underdevelopment of Raschkow’s plexus [12]. EPT is associated with an increased threshold value in teeth with open apices, which frequently results in increased rates of false-negative results [40]. HPT have shown limited value because of inconsistent responses, whereas CPT with CO_2_ ice proved to offer consistently more reliable results [42]. Under these conditions, it is considered that during root development, the treatment strategy must not be based on a negative response to pulp testing, but rather on radiographic and symptomatic assessment [39]. Furthermore, intraoperative assessment of the pulp condition by direct visualization is currently a valuable clinical aid, given the expanding use of dental operating microscopy [43]. The intraoperative assessment of pulpal bleeding, tissue color, and consistency guide the confirmation or denial of the diagnosis and allow clinicians to implement the most appropriate treatment approach [44].

## 5. Vitality Pulp Tests

Vitality pulp tests are used for the assessment of various parameters that characterize the vascular supply of the dental pulp, which is the true determinant of the pulp’s vitality. Laser Doppler flowmetry (LDF), transmitted laser light (TLL), laser speckle imaging (LSI), pulse oximetry (PO), transmitted light plethysmography (TLP), and dual wavelength spectrophotometry (DWS) are pulp testing methods based on optical technology [45]. They are completely noninvasive, painless, and objective (require no subjective response from the patient) [46]. One of the drawbacks that all light-transmitting devices used for pulp testing have in common is that they are limited to teeth containing pulp tissue within the coronal part of the tooth [7].

PO and LDF are the most studied vitality pulp tests, with the best clinical results among the pulp testing methods [10]. TLP and TLL were developed for pulp testing in an attempt to improve some of the limitations of PO and LDF respectively, but with relative success, as they come with limitations of their own [47]. DWS has been studied in-vitro with good results and served as a base for the development of pulse-oximetry [48].

### 5.1. Laser Doppler Flowmetry

The laser Doppler flowmetry technique uses a laser source aimed at the pulp tissue. The backscattered reflected light from circulating blood cells is Doppler-shifted and has a different frequency to the static surrounding tissues. The total backscattered light is processed to produce an output signal, commonly recorded as the concentration and velocity (flux) of cells, using an arbitrary term “perfusion units” (PU) [3]. The laser light can penetrate densely for up to 4 mm in depth and less densely for up to 13 mm [49]. Therefore, pulpal blood flow measurement using LDF should be carried out with proper isolation, to rule out signal contamination from the gingival tissue [49]. Isolation means used in different studies are opaque rubber dam, polyvinyl siloxane splint (also used for stabilization of the probe on the tooth [50]) alone, or in combination with light cure periodontal liquid dam [51]. The ideal position for the probe tip is 2–3 mm from the gingival margin [52]. Routine use of LDF is hindered by the method being too expensive and requiring technique-sensitive equipment [7]. Physical activity was found to significantly influence the results of LDF measurements of the pulpal blood flow, when employed before the procedure [53]. Furthermore, there are conflicting results reported in the literature. In a study on traumatized teeth, Belcheva et al. concluded that LDF is a useful monitoring tool for the revascularization of traumatized teeth and a reliable objective diagnostic indicator of pulp vitality [54]. A case report revealed the successful follow-up of pulpal status using LDF and EPT after vital pulp therapy performed in a dens invaginatus [55]. On the other hand, a study by Ghouth et al. comparing the diagnostic accuracy of LDF to CPT with ethyl chloride and EPT in permanent incisors of 8 to 16 year-old patients concluded that LDF was unable to differentiate between teeth with vital and non-vital pulps and expressed reluctance about the clinical use of LDF technology in the current form, especially in children [56]. Komatsu et al. found that in children, the pulpal blood flow of primary teeth evaluated by LDF showed a tendency to decrease with age, which they related to the morphological changes in the blood vessels in the pulp [57]; in contrast Karayilmaz et al. reported an increase of the pulpal blood flow in primary teeth with age, attributed to the progressive apical enlargement caused by physiological root resorption [58].

### 5.2. Transmitted Laser Light

Transmitted laser light is an experimental variation of LDF. Unlike LDF, in which the emitted light is backscattered to a single probe placed on the buccal side of the tooth, the TLL device uses two probes for sending and receiving the signal, placed on the buccal and palatal/lingual sides of the tooth. Sasano et al. [47] suggest that TLL is able to overcome the main drawbacks of LDF related to recording signals of non-pulpal origin and being sensitive to oscillations connected to heart rate. Although the method brings a much-needed improvement, it is still technique-sensitive and hard to implement in routine practice in the current form.

### 5.3. Laser Speckle Imaging

Laser speckle imaging by transillumination of the tooth was investigated in a few in vitro models [59,60]. It enables the differentiation between the absence and presence of perfusion and has the advantage that it is relatively insensitive to the angle of incidence of the laser light, which suggests that the precise positioning of an eventual probe design in the mouth is unnecessary to enable the accurate interrogation of the pulpal chamber for the presence of blood flow [59]. Recently, an upgraded version of LSI—the polarized laser speckle contrast imaging (LSCI) system, has shown improvement in pulpal blood flow monitorization, being able to detect a wider range of blood flow velocity and smaller changes of blood flow [61]. In-vivo studies are needed to test these experimental methods.

### 5.4. Pulse-Oximetry

Pulse oximetry is used to evaluate vascularization by determining the oxygen saturation level in the circulating arterial blood. It has numerous medical applications in different medical fields, through the use of finger, toe, foot and ear PO probes [62]. The PO probe consists of two light-emitting diodes (LEDs), one transmitting red light energy (660 nm) and the other transmitting infrared light energy (940 nm), and a photodetector diode connected to a signal-processing unit. Oxygenated and deoxygenated hemoglobin absorb different amounts of red and infrared lights. The oximeter correlates this information with the known absorption curves for oxygenated and deoxygenated hemoglobin to determine the oxygen saturation levels [63]. In dentistry, PO pulp testing is carried out using modified sensor holders [64,65,66], as a dedicated pulse-oximeter for dental use is not yet available [67]. Based on a simple and inexpensive technology, the pulse oximeter is an affordable, reliable, and easily available equipment for an average dental office [7,62]. The technique implies the application of the double-ended sensor (transmitter-receptor) onto the tooth and maintaining a fixed position during monitoring. Two essential requirements for a PO sensor dedicated to dental use are: to be shaped in a way that allows the transmitter and the detector to be parallel while fixed onto the tooth, and to be self-mounting (rather than hand-held), in order to provide more reliable readings. Due to its atraumatic approach, PO is suitable for pediatric use [63], but there are a few limitations of the technique that include the inability of the device to provide data for very small primary teeth (like the inferior incisors) and erupting teeth [68]. Moreover, there is a lack of evidence on how different systemic and oral pathologies may affect the pulp’s oxygen saturation levels [67] and how differing optical properties of the teeth may influence results [69]. Despite these drawbacks, PO has shown consistent good sensibility and specificity in different clinical situations [45,67]. It proved to be an effective tool in diagnosing different pulpal pathologies (especially pulp necrosis) [70] in primary teeth, as well as in immature and mature permanent teeth [45], and also in monitoring recently traumatized teeth, with a considerably better accuracy than sensibility tests [71].

### 5.5. Transmitted Light Photoplethysmography

Transmitted-light photoplethysmography is another optical technology is capable of assessing the pulpal blood flow. It was developed for pulp testing in an attempt to improve pulse oximetry by adding a light with a shorter wavelength [3]. TLP uses a light source to illuminate the tissues and a photo-detector to measure the changes in light intensity in relation with the changes in perfusion. Only specific wavelengths of light are absorbed by haemoglobin, and the remaining light passes through the tooth and is detected by a receptor. In immature permanent teeth, significant relationship between the TLP signal and the status of root formation was found [72]. Studies suggest that TLP has the advantage of less signal contamination derived from the surrounding tissues [72,73].

### 5.6. Dual Wavelength Spectrophotometry

Dual wavelength spectrophotometry is another optical, non-invasive pulp-testing method, similar to PO and TLP, which detects the presence or absence of oxygenated blood, and does not rely on the pulsatile circulation. It uses visible light at two different wavelengths—760 and 850 nm, filtered and guided to the tooth by fiberoptics [74]. Despite the simplicity of the technique, studies on pulp testing using DWS are scarce. The main limitation of DWS is that it detects only the presence of hemoglobin, not the circulation of blood [28].

## 6. Pulp Testing of Traumatized Teeth

It is well acknowledged that traumatized teeth might have no response to a stimulus for a variable period of time following injury, while vitality is maintained. It can take a minimum of 4–6 weeks for the pulp to recover and regain its sensory activity [75]. The likely causes behind this transient loss of pulp sensibility include pressure or tension on the nerve fibers, blood vessel rupture, and ischaemic injury [76]. In pediatric trauma cases, patient compliance might be also challenging, because of the young age of the patient and the distress felt after a traumatic event, consequently leading to false results of the sensibility tests [40]. Furthermore, the International Association of Dental Traumatology guidelines recommend against the use of sensibility tests in primary teeth, due to their lack of reliability [17]. In such cases, vitality tests (PO, LDF) proved to be extremely useful diagnostic tools to assess the pulp status, for both treatment strategy and follow-up [17,39,71,77,78].

## 7. Comparative Studies

A comparative study between different CPT agents, EPT, and LDF concluded that LDF was the most reliable and accurate among the tests, but most time-consuming. On the other hand, the accuracy and repeatability of CPT and EPT results varied between them, depending on the CPT agent that was used. Multiple studies revealed the superiority of CO_2_ snow and refrigerant sprays over ethyl chloride and ice [42].

Comparative studies led by Sharma et al. and Samuel et al. on PO versus CPT and EPT in primary teeth, and immature and mature permanent teeth indicate an equal accuracy between PO and CPT, and the inferiority of EPT. They suggest that PO can be used as a routine method for assessing the pulp vitality of the afore-mentioned dental categories [23,45]. In a similar study, Janani et al. found PO to be the most accurate dental pulp test in permanent teeth in need of endodontic treatment, followed by CPT and EPT [79]. Karayilmaz et al. compared PO, LDF, and EPT in the mature permanent incisors of adolescent patients and concluded that LDF is a more reliable and effective method than PO and EPT [80].

A study on traumatized permanent incisors compared the results of EPT, CPT, and PO over a 6-month period and found that PO gave positive vitality readings that remained constant throughout the whole study period in all patients, whereas the proportion of teeth showing a positive responsiveness in CPT (tetrafluoroethane refrigerant spray) and EPT increased gradually from no teeth showing responsiveness on day 0 to 94.11% teeth at 3 months [81].

Two systematic reviews of studies investigating the diagnostic accuracy of the CPT, HPT, EPT, LDF, and PO revealed that LDF and PO had a higher percentage of teeth correctly identified as vital or nonvital (accuracy) compared to thermal and electrical pulp testing [10], with HPT being the least accurate among the testing methods [9]. On the other hand, a systematic review of LDF studies concluded that, despite the higher reported sensitivity and specificity of LDF in assessing pulp blood flow, these data are based on studies with a high level of bias and serious shortfalls in study designs and the literature is lacking information about the cut-off ratios over which a tooth can be diagnosed as healthy [82].

## 8. Guideline for Using Pulp Tests in Pediatric Clinical Practice

**Figure 2:**
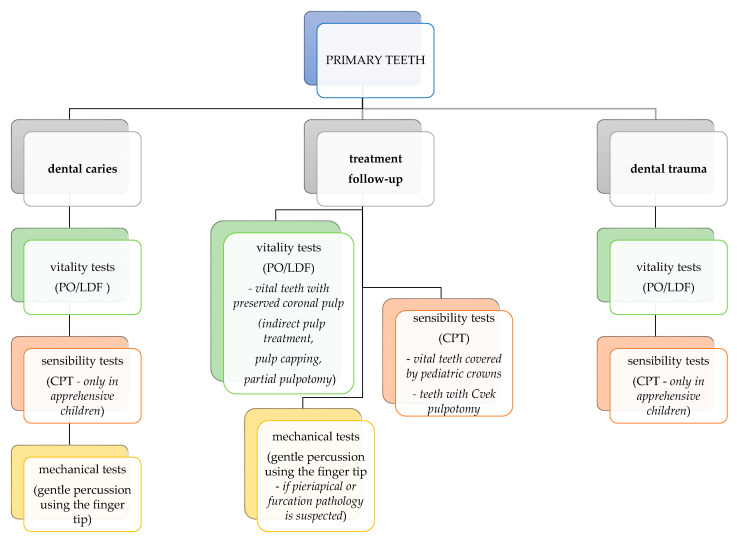
Pulp test selection chart for different clinical situations in primary teeth.

Considering the advantages and disadvantages of the pulpal tests detailed above, only a few are applicable in pediatric dentistry. In young children, mechanical tests are not recommended due to their invasive nature and potential to trigger disruptive behavior in otherwise cooperative patients. The only exception is the modified percussion technique using only gentle finger pressure, which is acceptable provided that adequate behavior management is ensured during the procedure. Regarding sensibility tests, the use of thermal testing in primary teeth is still largely debated in today’s literature. We advise the use of CPT in primary teeth to be considered as an alternative when vitality testing is inconclusive or not available, and we recommend against the use of HPT and EPT in primary teeth. CPT is to be applied only in children who can understand the instructions and provide an adequate response to the stimuli. A guide for pulp test selection in primary teeth for different clinical situations is presented in Figure 2.

**Figure 3:**
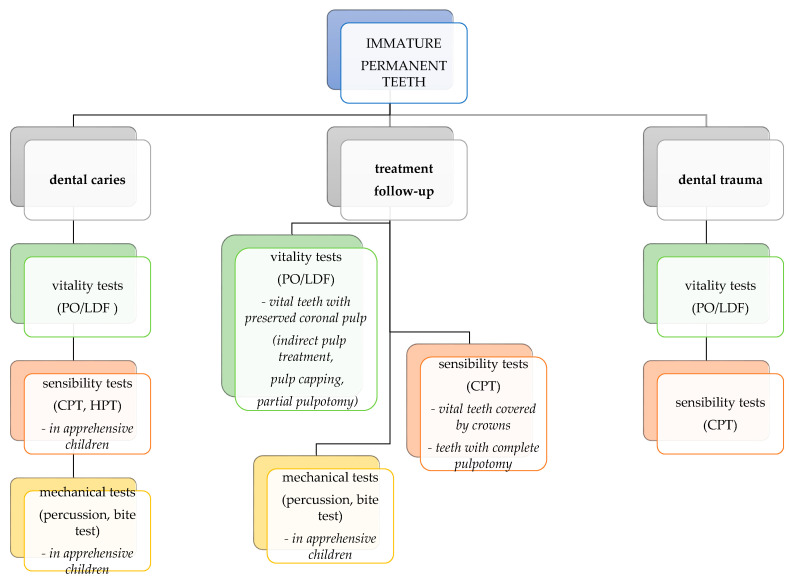
Pulp test selection chart for different clinical situations in immature permanent teeth.

Regarding immature permanent teeth, vitality tests have the highest accuracy and are especially useful in trauma cases. Thermal and mechanical tests are indicated as a second option in older, apprehensive children, whereas EPT is not considered reliable in immature permanent teeth. A guide for pulp test selection in immature permanent teeth for different clinical situations is presented in Figure 3. Sensibility tests such as selective anaesthesia and test cavity should not be used in children.

## 9. Concluding Remarks and Future Perspectives

Clinical pulpal diagnosis is a field where more high-quality studies are needed for the development of practical and reliable tests to aid the clinical decision-making process. For primary and immature permanent teeth, sensibility tests are generally considered unreliable by the majority of authors, but they can be used in particular cases, in conjunction with other clinical diagnostic aids. A multitude of systematic reviews on pulp testing methods [7,9,10,71,82] have highlighted the low quality of evidence found in the existing studies on this topic, owed to the high risk of bias and deficiency in the research design. However, the data consistently proves the diagnostic accuracy and superiority of vitality tests when compared with sensibility tests, in all clinical scenarios. Among the vitality testing methods, pulse oximetry seems to have a series of advantages which make this method suitable for pediatric dental use, and the results of clinical studies are promising. Although the technique is not perfected for routine clinical use yet, it is an encouraging and expanding study area.

## Data Availability

Not applicable.
